# The causal effects of thyroid function and lipids on cholelithiasis: A Mendelian randomization analysis

**DOI:** 10.3389/fendo.2023.1166740

**Published:** 2023-03-29

**Authors:** Junhong Chen, Hao Zhou, Hengwei Jin, Kai Liu

**Affiliations:** Department of Hepatobiliary and Pancreatic Surgery II, General Surgery Center, The First Hospital of Jilin University, Changchun, China

**Keywords:** thyroid function, lipid metabolism traits, cholelithiasis, Mendelian randomization, mediation effects

## Abstract

**Objective:**

To investigate the relationship between function of thyroid, lipids, and cholelithiasis and to identify whether lipids mediate the causal relationship between function of thyroid and cholelithiasis.

**Methods:**

A Mendelian randomization (MR) study of two samples was performed to determine the association of thyroid function with cholelithiasis. A two-step MR was also performed to identify whether lipid metabolism traits mediate the effects of thyroid function on cholelithiasis. A method of inverse variance weighted (IVW), weighted median method, maximum likelihood, MR-Egger, MR-robust adjusted profile score (MR-RAPS) method, and MR pleiotropy residual sum and outlier test (MR-PRESSO) methods were utilized to obtain MR estimates.

**Results:**

The IVW method revealed that FT4 levels were correlated with an elevated risk of cholelithiasis (OR: 1.149, 95% CI: 1.082–1.283, *P* = 0.014). Apolipoprotein B (OR: 1.255, 95% CI: 1.027–1.535, *P* = 0.027) and low-density lipoprotein cholesterol (LDL-C) (OR: 1.354, 95% CI: 1.060–1.731, *P* = 0.016) were also correlated with an elevated risk of cholelithiasis. The IVW method demonstrated that FT4 levels were correlated with the elevated risk of apolipoprotein B (OR: 1.087, 95% CI: 1.019–1.159, *P* = 0.015) and LDL-C (OR: 1.084, 95% CI: 1.018–1.153, *P* = 0.012). Thyroid function and the risk of cholelithiasis are mediated by LDL-C and apolipoprotein B. LDL-C and apolipoprotein B had 17.4% and 13.5% of the mediatory effects, respectively.

**Conclusions:**

We demonstrated that FT4, LDL-C, and apolipoprotein B had significant causal effects on cholelithiasis, with evidence that LDL-C and apolipoprotein B mediated the effects of FT4 on cholelithiasis risk. Patients with high FT4 levels should be given special attention because they may delay or limit the long-term impact on cholelithiasis risk.

## Introduction

Cholelithiasis, a prevalent condition affecting approximately 10–20% of the global adult population, has experienced a recent upsurge in incidence (1). The association between cholelithiasis and the onset of the gallbladder, pancreatic, and colorectal cancers is well established (2). While the majority of affected adults remain asymptomatic, the economic and societal burdens of cholelithiasis can be substantial in the event of symptomatology or complications (3, 4).

Cholelithiasis remains a prevalent gastrointestinal disorder for which the pathophysiology is still unknown. Recent clinical observational studies have shed light on a potential association between thyroid function and cholelithiasis. Notably, a study encompassing a cohort of 3,749 subjects aged 20 to 79 demonstrated an independent correlation between cholelithiasis and elevated serum thyroid stimulating hormone (TSH) levels (5). Furthermore, patients with cholelithiasis exhibited a significantly higher prevalence of both subclinical and clinical hypothyroidism (6). However, conventional observational studies are limited in their ability to determine causal effects and account for potential confounding factors.

Several convincing studies state a positive correlation between high cholesterol levels and the development of cholelithiasis (7). Furthermore, serum FT4 levels are positively correlated with total triglycerides (TG) and LDL-C (8). It was also identified that lipid metabolism traits might mediate the causal effects of thyroid function on cholelithiasis.

Utilizing genetic variants as instrument variables (IVs), MR analysis has emerged as a powerful tool for determining the causal relationship between risk factors and diseases (9). Large-scale genome-wide summary association studies (GWAS) also allow for the systematic investigation of the causal effects of exposures on outcomes using MR (10). In the current investigation, MR analysis was used to evaluate the relationship between thyroid function, lipids, and cholelithiasis and to determine whether lipids mediate the causal effects of thyroid function on cholelithiasis.

## Materials and methods

### Study design and GWAS statistics source

The total effects were determined using a two-sample MR to evaluate the association between thyroid function and cholelithiasis. Another two-step MR was performed to assess whether lipid metabolism traits mediate the effects of thyroid function on cholelithiasis. First, we explore the relationship between function of thyroid and lipid metabolism traits. Second, we investigated the effects of lipid metabolism traits on cholelithiasis risk.

We retrieved the GWAS thyroid function summary data from the ThyroidOmics Consortium, which was formed to study the determinants and effects of thyroid disorders and thyroid function (11). In a meta-analysis, analyses of TSH comprised information from 22 different cohorts comprising 54,288 individuals, FT4 analyses had data from 19 cohorts with 49,269 individuals, hypothyroidism data from 53,423 individuals, and hyperthyroidism data from 51,823 subjects (11). The UK Biobank (UKB) provided summary statistical data for lipids (LDL-C, apolipoprotein B, and TG) (12). The sample size for LDL-C, apolipoprotein B, and TG was 440,546, 439,214, and 441,015, respectively. The UKB data came from a prospective cohort study that enrolled over 500,000 males and females (40–69 years old at baseline) between 2006 and 2010 (13). FinnGen Biobank of European ancestry provided the GWAS associated with cholelithiasis (19,023 cases and 195,144 controls). FinnGen is a large public-private partnership that aims to collect and analyze genomic and health data from 500,000 participants in Finnish biobanks. **Table 1** contains detailed information.

**Table 1:** Details of GWAS included in MR analyses.

Traits	Consortia	Ethnicity	Sample size
Cholelithiasis (n, %)	FinnGen Biobank	European	214,167
FT4 (mmol/L)	The ThyroidOmics Consortium	European	49,269
TSH (mmol/L)	The ThyroidOmics Consortium	European	54,288
Hyperthyroidism (n, %)	The ThyroidOmics Consortium	European	51,823
Hypothyroidism (n, %)	The ThyroidOmics Consortium	European	53,423
LDL-C (mmol/L)	UK Biobank	European	440,546
Apolipoprotein B (mmol/L)	UK Biobank	European	439,214
Triglyceride (mmol/L)	UK Biobank	European	441,016

### Selection of genetic instrumental variables

We identified single-nucleotide polymorphisms (SNPs) with genome-wide significance (*P*< 5 × 10^−8^), linkage disequilibrium (LD), and an *r^2<^* 0.001 threshold within a 10,000 kb window (14). We used PhenoScanner, a genotype-to-phenotype cross-reference (www.phenoscanner.medschl.cam.ac.uk), to look for secondary phenotypes associated with the selected instruments. In the present study, when cholelithiasis was identified as the outcome, blood glucose, BMI, and cholecystitis were identified as confounding factors. The palindromic variants were removed for incompatible alleles. When the SNPs were unavailable in the outcomes GWAS datasets, proxy SNPs were used. The final IVs for the subsequent MR study consisted of the strictly chosen SNPs. F-statistic was calculated to assess the strength of the selected SNPs according to the following equation:


$$F=\frac{R^{2}\left(N-1-K\right)}{\left(1-R^{2}\right)K}$$

Where R^2^ is the portion of exposure variance explained by the IVs, N is the sample size, and K is the number of IVs. F-statistic ≧ 10 indicates no strong evidence of weak instrument bias.

### Replicative analysis

The Global Lipids Genetics Consortium (GLGC) was the source for the summary statistics of LDL-C and apolipoprotein B, while cholelithiasis was procured from the UK Biobank to serve as a replicative analysis.

### The proportion of mediation effects

The total effects of exposure on an outcome can be divided into indirect and direct effects (15). After adjusting for LDL-C, apolipoprotein B, and TG, MR revealed direct effects of thyroid function on cholelithiasis risk. The product method was used to calculate the indirect effects of lipid traits by multiplying the effects of thyroid function on lipid traits and the effects of lipid traits on cholelithiasis (16). The following equation was used to calculate the proportion of the mediation effects (17):


$$E\left(\%\right)=\frac{\sum_{k=1}^{K}\;\beta_{1}*\beta_{2k}}{\sum_{k=1}^{K}\;\beta_{3}+\beta_{1}*\beta_{2k}}$$

Where β1 represents the MR effects of thyroid function on mediator k by two-step MR, β2 represents the MR effects of mediator k on cholelithiasis risk by two-step MR, and β3 represents the MR effects of thyroid function on cholelithiasis risk by two-sample MR.

### Statistical analysis

For MR analysis, five different methods [inverse-variance weighted (IVW), weighted median, MR Egger, maximum likelihood, and MR-robust adjusted profile score (MR-RAPS)] were used. To estimate the causal effects, the IVW method combines the wald ratios of the causal effects of each SNP. Each IV in this method must fulfill the three MR assumptions, or the derived estimates may be biased in the case of horizontal pleiotropy (18, 19). Compared with other MR methods, the maximum likelihood method provides an estimator with the lowest standard error under almost all conditions (20). MR-RAPS has been performed to model the random-effects distribution of pleiotropic genetic variation effects (21).

The MR-Egger analysis was used to evaluate the potential pleiotropic effects of SNP. The intercept *P*-value indicated if horizontal pleiotropy interfered with the MR estimates in MR Egger analysis. There was significant pleiotropy if the intercept *P*-value< 0.05. Cochrane’s Q value was utilized to evaluate the heterogeneity among SNPs in IVW estimates. By excluding each SNP from the analysis, leave-one-out sensitivity analyses can be used to decide whether a single SNP has a significant effect on the overall result. The outlier variants were determined *via* the MR pleiotropy residual sum and outlier test (MR-PRESSO). The funnel plot was used to demonstrate the symmetrical distribution of the selected SNPs. A *P*-value< 0.05 was regarded as significant.

The statistical analyses were conducted utilizing the TwoSampleMR R package (version 0.5.5), MR-RAPS (version 1.0), and MR-PRESSO (version 1.0) with R software 4.1.2.

## Results

### MR analysis between thyroid function and cholelithiasis

**Figure 1:**
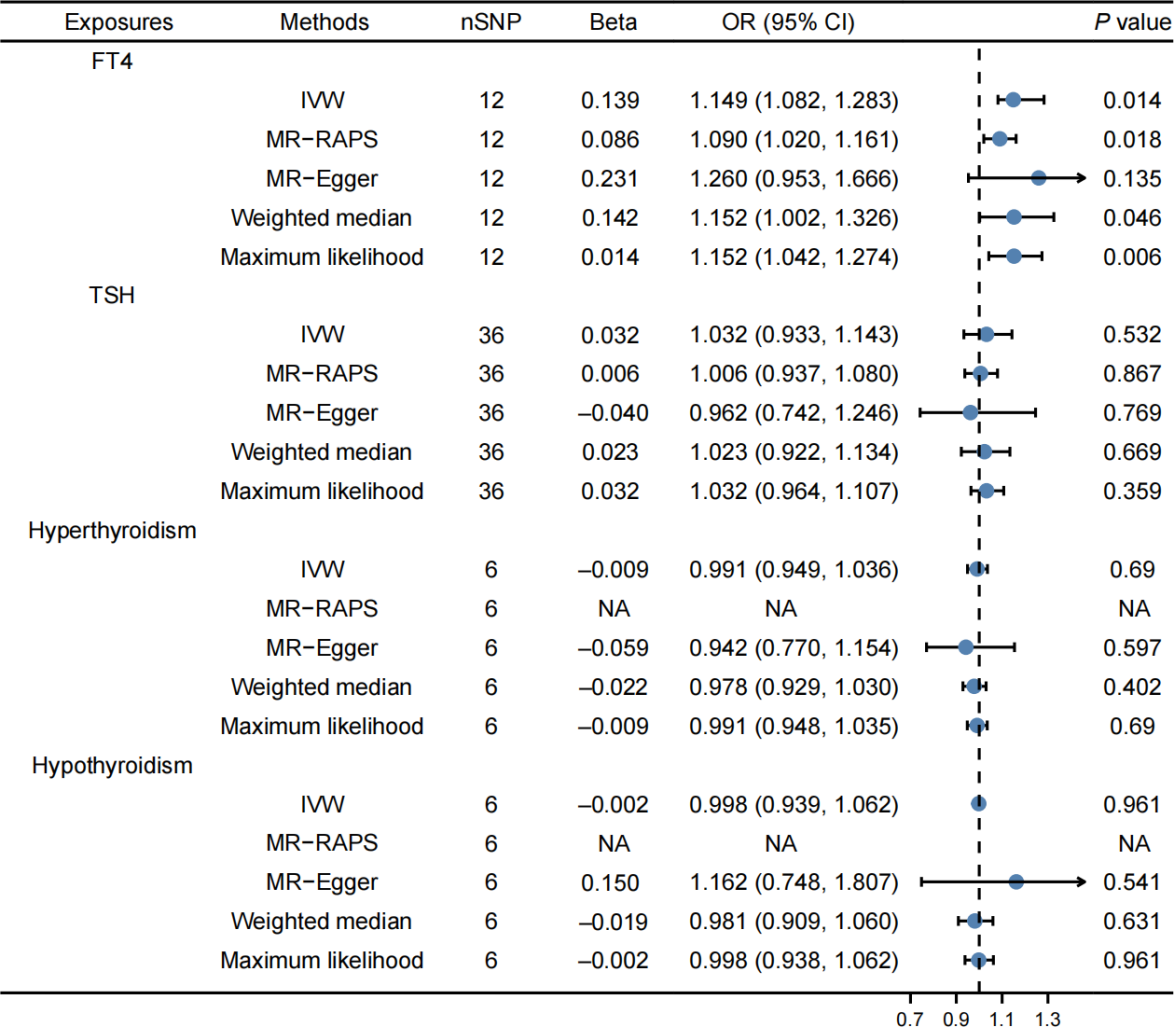
Effects of thyroid function on cholelithiasis. FT4 levels were correlated with an elevated risk of cholelithiasis, while TSH, hyperthyroidism, and hypothyroidism were not related to the risk of cholelithiasis.

Finally, 12 SNPs were used as IVs for FT4, 36 SNPs for TSH, 6 SNPs for hyperthyroidism, and 6 SNPs for hypothyroidism. **Supplementary Tables 1–4** provided detailed information. In our study, F-statistic for each instrument-exposure association ranged from 19.867 to 37.321, demonstrating the less possibility of weak instrumental variable bias in the final results (**Supplementary Table 11**). The IVW method revealed that FT4 levels were correlated with an elevated risk of cholelithiasis (OR: 1.149, 95% CI: 1.082–1.283, *P* = 0.014, **Figure 1**). Weighted median (OR: 1.152, 95% CI: 1.002–1.326, *P* = 0.046), maximum likelihood (OR: 1.152, 95% CI: 1.042–1.274, *P* = 0.006), and MR-RAPS (OR: 1.090, 95% CI: 1.020–1.161, *P* = 0.018) indicated consistent results, while MR Egger illustrated negative results. In addition, the IVW method revealed that TSH, hyperthyroidism, and hypothyroidism were not related to the risk of cholelithiasis (**Figure 1**). Similar results are obtained through the implementation of alternative methodologies and the use of replicative analyses, as demonstrated by the findings presented in **Supplementary Table 12**.

The MR-PRESSO method also revealed that high FT4 levels were correlated with an elevated risk of cholelithiasis, and no outlier SNPs were recognized (**Table 2**). The present MR analysis revealed no horizontal pleiotropy and heterogeneity (**Supplementary Table 10**). Moreover, the stability of the results was indicated by the symmetrical shape of the funnel plot, as presented in **Supplementary Figure 1**. The findings of additional analyses related to thyroid function using MR are summarized in **Table 2**; **Supplementary Table 10**. Furthermore, the use of the leave-one-out sensitivity analysis demonstrated the robustness of the results, as depicted in **Supplementary Figure 6**.

**Table 2:** MR-PRESSO estimates between exposures and outcomes.

Exposures	Outcomes	Raw estimates			Outlines corrected estimates		
		N	Beta	P value	N	Beta	P value
FT4	Cholelithiasis	12	0.138	0.017	12	NA	NA
TSH	Cholelithiasis	36	0.032	0.542	35	0.010	0.772
Hyperthyroidism	Cholelithiasis	6	0.002	0.903	6	NA	NA
Hypothyroidism	Cholelithiasis	6	–0.002	0.950	6	NA	NA
LDL-C	Cholelithiasis	155	0.167	0.021	149	0.185	0.011
Triglyceride	Cholelithiasis	277	0.013	0.795	260	0.035	0.358
Apolipoprotein B	Cholelithiasis	179	0.299	0.025	163	0.143	<0.001
FT4	LDL-C	12	0.721	0.014	12	NA	NA
FT4	Apolipoprotein B	12	0.071	0.003	12	NA	NA

### MR analysis between lipids and cholelithiasis

**Figure 2:**
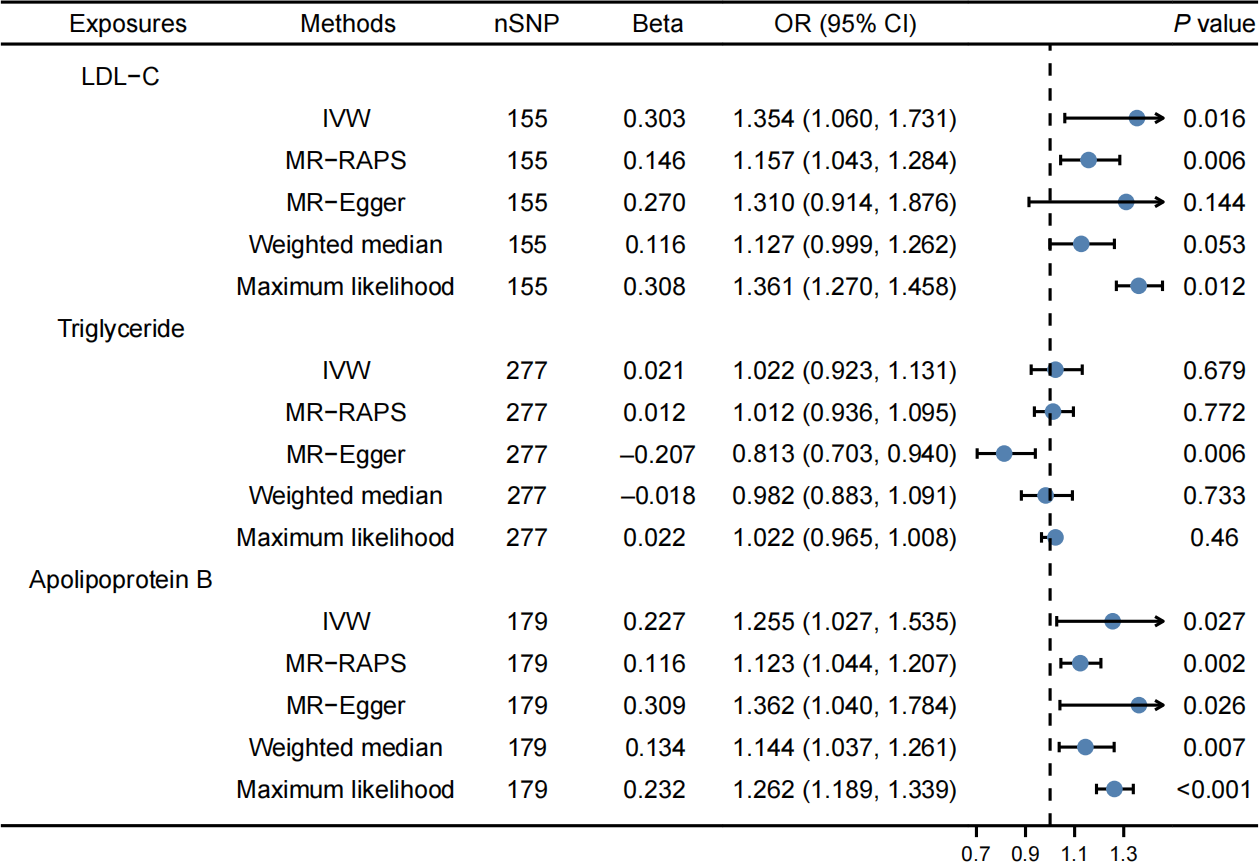
Effects of lipids on cholelithiasis. LDL-C and apolipoprotein B levels were correlated with an elevated risk of cholelithiasis, while triglyceride was not related to the risk of cholelithiasis.

Finally, 155 SNPs were used as IVs for LDL-C, 277 SNPs for TG, and 179 SNPs for apolipoprotein B. **Supplementary Tables 5–7** contain detailed information. The IVW method demonstrated a significant correlation between LDL-C and an elevated risk of cholelithiasis (OR: 1.354, 95% CI: 1.060–1.731, *P* = 0.016, **Figure 2**). The MR-PRESSO method also revealed that LDL-C was linked to an elevated risk of cholelithiasis (**Table 2**). The result was consistent after the outliners were removed. In this MR study, no horizontal pleiotropy was observed, but there was heterogeneity (**Supplementary Table 10**). The IVW method revealed that apolipoprotein B was correlated with an elevated risk of cholelithiasis (OR: 1.255, 95% CI: 1.027–1.535, *P* = 0.027, **Figure 2**). However, there was no correlation between TG and cholelithiasis risk. Moreover, the results of the study were observed to be stable based on the symmetrical funnel plots (**Supplementary Figures 2**, **3**) and the leave-one-out method (**Supplementary Figures 7**, **8**). Additionally, the replicative analysis further confirmed the consistency of the findings (**Supplementary Table 12**).

### MR analysis between thyroid function and lipids

**Figure 3:**
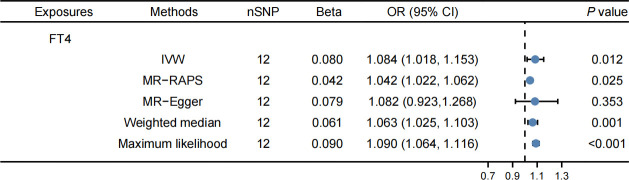
Effects of FT4 on LDL-C. FT4 levels were correlated with an elevated risk of LDL-C.

**Figure 4:**
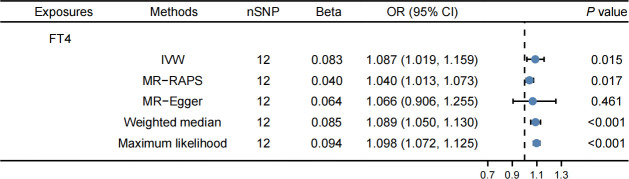
Effects of FT4 on apolipoprotein B. FT4 levels were correlated with an elevated risk of apolipoprotein B.

**Supplementary Tables 8, 9** contain detailed information on IVs. The IVW method revealed that FT4 levels were correlated with an elevated risk of LDL-C (OR: 1.084, 95% CI: 1.018–1.153, *P* = 0.012, **Figure 3**) and apolipoprotein B (OR: 1.087, 95% CI: 1.019–1.159, *P* = 0.015, **Figure 4**). The MR-PRESSO method produced consistent results, with no outlier SNPs identified (**Table 2**). In the present MR analysis, there was no horizontal pleiotropy, but there was heterogeneity (**Supplementary Table 10**).

In addition, the symmetrical nature of the funnel plots provides evidence for the stability of the findings as demonstrated in **Supplementary Figures 4**, **5**. The leave-one-out approach further reinforces the reliability of the results, as evidenced by the consistency of the outcomes shown in **Supplementary Figures 9**, **10**. Additionally, the replicative analysis, as illustrated in **Supplementary Table 12**, yields comparable findings, further consolidating the robustness of the results.

### The proportion of the mediatory effects of apolipoprotein B and LDL-C

The present analysis revealed that apolipoprotein B and LDL-C mediated the effects of thyroid function on cholelithiasis risk. LDL-C and apolipoprotein B had 17.4% and 13.6% of the mediatory effects, respectively.

## Discussion

In the current study, Mendelian randomization was employed to investigate the causal associations between thyroid function and lipids in relation to cholelithiasis. The results indicate a positive association between FT4 levels and cholelithiasis risk. Additionally, elevated levels of LDL-C and apolipoprotein B were also significantly associated with an increased risk of cholelithiasis. MR analysis revealed that LDL-C and apolipoprotein B accounted for 17.4% and 13.5% of the mediatory effects, respectively. These findings provide important insights into the role of thyroid function and lipid metabolism traits in the pathogenesis of cholelithiasis, which may have implications for developing preventive and therapeutic strategies for this disease.

In prior studies, observational analyses have predominantly established a correlation between thyroid function and the presence of cholelithiasis. As reported by J. Inkinen in previous research, a significant association was detected between the occurrence of common bile duct stones and pre-existing hypothyroidism (22). A study was conducted on a sample of 3,749 individuals aged between 20 and 79 years, which revealed a statistically significant and independent association between increased serum thyrotropin (TSH) levels and cholelithiasis in males (5). However, no such relation was identified in the female population. In contrast, some studies have suggested a greater susceptibility of women to both cholelithiasis and thyroid disorders (23). Animal models have also indicated that hyperthyroidism may be a predisposing factor for cholelithiasis. Furthermore, a Chinese researcher has proposed that dysfunction of the thyroid, including both hyperthyroidism and hypothyroidism, can promote the formation of gallstones through various pathways (24). However, the existence of residual confounding, reverse causation, or both, has been raised as potential explanations for the observed associations. In the present study, we found that neither TSH levels, hyperthyroidism, nor hypothyroidism were significantly related to the risk of cholelithiasis. Notably, this is the first MR analysis to demonstrate that elevated FT4 levels confer an increased risk of cholelithiasis.

Although various investigations have been performed to clarify the association between lipids and cholelithiasis, the findings remain controversial. Several convincing studies revealed a positive association between high cholesterol levels and cholelithiasis (2). In a case-control study, Fu et al. found that increased serum LDL-C and apolipoprotein B were an index of cholesterol stones (25). However, Tang et al. identified that apolipoprotein A, B, high serum HDL, and lower LDL are risk factors for cholelithiasis in a study with 109 sample sizes. The studies mentioned above had limited sample sizes. LDL-C and apolipoprotein B were also correlated with an elevated risk of cholelithiasis in our large-scale MR study.

The underlying mechanisms of thyroid function and cholelithiasis remain unknown. Cholelithiasis can be caused by various factors, considering how thyroid hormones affect the balance of cholesterol, the amount of bile produced, biliary secretion, and motility of the gallbladder (26). Thyroid hormones have been shown to influence enterohepatic circulation and detoxification (27–29), as well as nuclear receptor-mediated LITH gene expression (2, 30–33). Thyroid dysfunction and lipid homeostasis were two other underlying mechanisms. We identified that LDL-C and apolipoprotein B mediate the effects of thyroid function on the cholelithiasis risk. LDL-C and apolipoprotein B had 17.4% and 13.5% of the mediatory effects, respectively. Reduced bile acid production by the conventional (CYP7A1 and CYP8B1) and alternative (CYP27A1) pathways were found in an *in vitro* research on primary human hepatocytes (34). It revealed that hyperthyroidism could cause a disturbance in the composition of bile through dysregulation of lipid homeostasis.

The present study has several strengths. First, it was the first MR to examine how lipids and thyroid function affect cholelithiasis using large-scale GWAS from UKB, Finngen Biobank, and the ThyroidOmics Consortium. Second, because the IVs we selected were located on a different chromosome, any possible gene-gene interaction may have few effects on the predicted value (35). Third, we used several stable methods to obtain the MR effects, such as MR-PRESSO and MR-RAPS. Furthermore, we assessed horizontal pleiotropy. Finally, using the two-step MR analysis, we identified that LDL-C and apolipoprotein B acted as mediators of the causal pathway from FT4 levels to cholelithiasis risk.

The present study has some limitations. First, there was potential heterogeneity due to differences in health status, age, or gender. Second, all participants were of European ancestry, which may restrict the applicability of the results to other races and ethnicities. Third, any potential nonlinear relationships or stratification effects result from the GWAS data. Fourth, TSH and FT4 levels were obtained from different cohorts which may have an impact on the reliability of the results. Finally, because confounding and mediation cannot be statistically differentiated, mediation analysis was critically dependent on the accurate characterization of the causal relationships (36).

## Conclusion

In conclusion, we demonstrated that FT4, LDL-C, and apolipoprotein B had significant causal effects on cholelithiasis, with evidence that the LDL-C and apolipoprotein B mediated the effects of FT4 on cholelithiasis risk. Patients with high FT4 levels should be given special attention because they may delay or limit the long-term impact on cholelithiasis risk.

## Data availability statement

The original contributions presented in the study are included in the article/**Supplementary Material**. Further inquiries can be directed to the corresponding author.

## Ethics statement

Ethical review and approval was not required for the study on human participants in accordance with the local legislation and institutional requirements. Written informed consent for participation was not required for this study in accordance with the national legislation and the institutional requirements.

## Author contributions

All authors listed have made a substantial, direct, and intellectual contribution to the work and approved it for publication.

## Supplementary material

Supplementary Figure 1 Funnel plot of the association between FT4 and cholelithiasis.

Supplementary Figure 2 Funnel plot of the association between LDL-C and cholelithiasis.

Supplementary Figure 3 Funnel plot of the association between apolipoprotein B and cholelithiasis.

Supplementary Figure 4 Funnel plot of the association between FT4 and LDL-C.

Supplementary Figure 5 Funnel plot of the association between FT4 and apolipoprotein B.

Supplementary Figure 6 Leave-one-out sensitivity analysis of the association between FT4 and cholelithiasis.

Supplementary Figure 7 Leave-one-out sensitivity analysis of the association between LDL-C and cholelithiasis.

Supplementary Figure 8 Leave-one-out sensitivity analysis of the association between apolipoprotein B and cholelithiasis.

Supplementary Figure 9 Leave-one-out sensitivity analysis of the association between FT4 and LDL-C.

Supplementary Figure 10 Leave-one-out sensitivity analysis of the association between FT4 and apolipoprotein B.
