# Supplementary figures and images for: The causal effects of thyroid function and lipids on cholelithiasis: A Mendelian randomization analysis
Source: Front Endocrinol (Lausanne). 2023 Mar 29;14:1166740. doi: 10.3389/fendo.2023.1166740 (PMC10090462; doi:10.3389/fendo.2023.1166740)

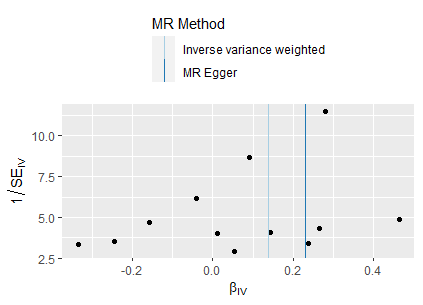

Supplement: Supplementary Figure 1 — Funnel plot of the association between FT4 and cholelithiasis. [file Image_1.png]

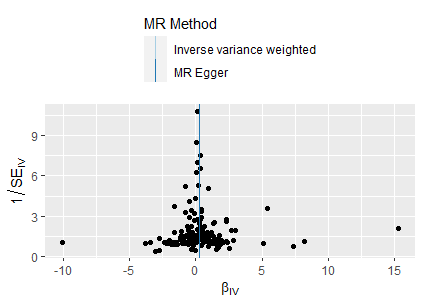

Supplement: Supplementary Figure 2 — Funnel plot of the association between LDL-C and cholelithiasis. [file Image_2.png]

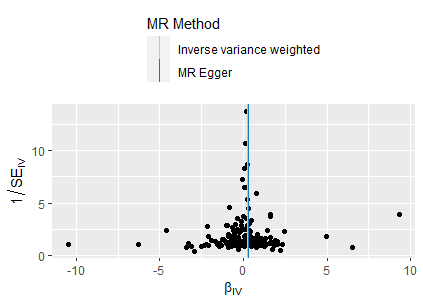

Supplement: Supplementary Figure 3 — Funnel plot of the association between apolipoprotein B and cholelithiasis. [file Image_3.png]

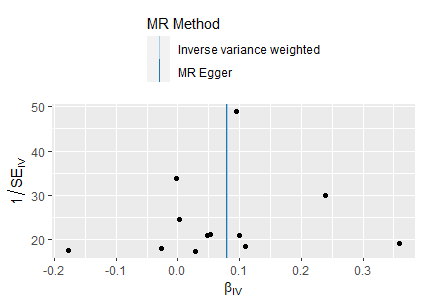

Supplement: Supplementary Figure 4 — Funnel plot of the association between FT4 and LDL-C. [file Image_4.png]

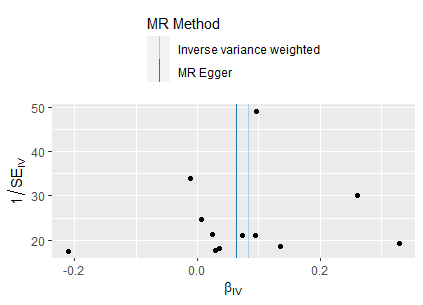

Supplement: Supplementary Figure 5 — Funnel plot of the association between FT4 and apolipoprotein B. [file Image_5.png]

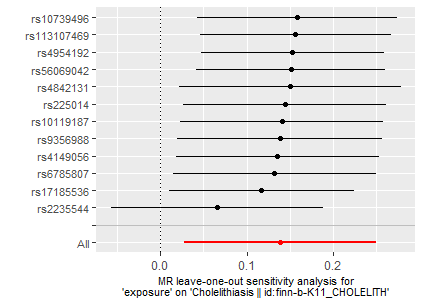

Supplement: Supplementary Figure 6 — Leave-one-out sensitivity analysis of the association between FT4 and cholelithiasis. [file Image_6.png]

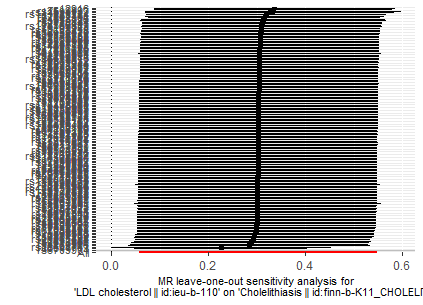

Supplement: Supplementary Figure 7 — Leave-one-out sensitivity analysis of the association between LDL-C and cholelithiasis. [file Image_7.png]

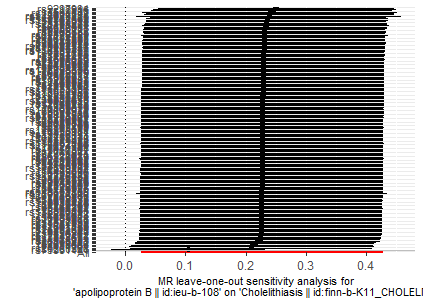

Supplement: Supplementary Figure 8 — Leave-one-out sensitivity analysis of the association between apolipoprotein B and cholelithiasis. [file Image_8.png]

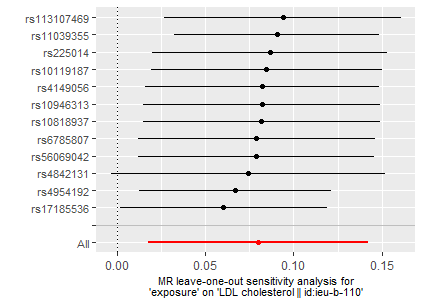

Supplement: Supplementary Figure 9 — Leave-one-out sensitivity analysis of the association between FT4 and LDL-C. [file Image_9.png]

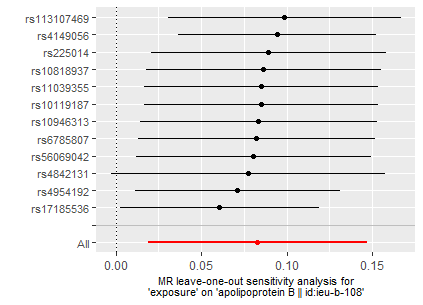

Supplement: Supplementary Figure 10 — Leave-one-out sensitivity analysis of the association between FT4 and apolipoprotein B. [file Image_10.png]
